# Delivery of primary health care to persons who are socio-economically disadvantaged: does the organizational delivery model matter?

**DOI:** 10.1186/1472-6963-13-517

**Published:** 2013-12-17

**Authors:** Simone Dahrouge, William Hogg, Natalie Ward, Meltem Tuna, Rose Anne Devlin, Elizabeth Kristjansson, Peter Tugwell, Kevin Pottie

**Affiliations:** 1 Department of Family Medicine, University of Ottawa, Ottawa, Canada; 2 C.T. Lamont Primary Health Care Research Centre, Bruyère Research Institute, Ottawa, Canada; 3 Institute of Population Health, University of Ottawa, Ottawa, Canada; 4 Department of Epidemiology and Community Medicine, University of Ottawa, Ottawa, Canada; 5 Department of Anthropology and Sociology, University of Ottawa, Ottawa, Canada; 6 Ottawa Hospital Research Institute, Ottawa, Canada; 7 Department of Economics, University of Ottawa, Ottawa, Canada; 8 Department of Psychology, University of Ottawa, Ottawa, Canada; 9 Centre for Global Health Research, Institute of Population Health, University of Ottawa, Ottawa, Canada; 10 Department of Medicine, The Ottawa Hospital, Ottawa, Canada

**Keywords:** Primary care, Health equity, Organizational models, Physician remuneration

## Abstract

**Background:**

As health systems evolve, it is essential to evaluate their impact on the delivery of health services to socially disadvantaged populations. We evaluated the delivery of primary health services for different socio-economic groups and assessed the performance of different organizational models in terms of equality of health care delivery in Ontario, Canada.

**Methods:**

Cross sectional study of 5,361 patients receiving care from primary care practices using Capitation, Salaried or Fee-For-Service remuneration models. We assessed self-reported health status of patients, visit duration, number of visits per year, quality of health service delivery, and quality of health promotion. We used multi-level regressions to study service delivery across socio-economic groups and within each delivery model. Identified disparities were further analysed using a t-test to determine the impact of service delivery model on equity.

**Results:**

Low income individuals were more likely to be women, unemployed, recent immigrants, and in poorer health. These individuals were overrepresented in the Salaried model, reported more visits/year across all models, and tended to report longer visits in the Salaried model. Measures of primary care services generally did not differ significantly between low and higher income/education individuals; when they did, the difference favoured better service delivery for at-risk groups. At-risk patients in the Salaried model were somewhat more likely to report health promotion activities than patients from Capitation and Fee-For-Service models. At-risk patients from Capitation models reported a smaller increase in the number of additional clinic visits/year than Fee-For-Service and Salaried models. At-risk patients reported better first contact accessibility than their non-at-risk counterparts in the Fee-For-Service model only.

**Conclusions:**

Primary care service measures did not differ significantly across socio-economic status or primary care delivery models. In Ontario, capitation-based remuneration is age and sex adjusted only. Patients of low socio-economic status had fewer additional visits compared to those with high socio-economic status under the Capitation model. This raises the concern that Capitation may not support the provision of additional care for more vulnerable groups. Regions undertaking primary care model reforms need to consider the potential impact of the changes on the more vulnerable populations.

## Background

Comprehensive and accessible primary health care is known to improve health outcomes and help reduce health inequities [1,2]. While the introduction of universal health care in Canada has gone a long way towards reducing inequities in health care accessibility, [3] significant gaps remain in the quality of care received by individuals of high versus low socio-economic status [4]. For example, individuals with low income or low education are less likely to have undergone cancer screening than wealthier and better educated individuals [5]. This is disconcerting, as socio-economically disadvantaged individuals have poorer self-rated health, [6] higher rates of obesity and alcohol consumption, [7,8] ischaemic heart disease, [9,10]. Type Two diabetes, and other chronic conditions, [11,12] and greater chances of premature mortality [13,14].

To address this disparity, Canadian [15] and international [16] policy recommendations emphasize the need for further investment in primary health care systems to improve effectiveness and fairness of access to health care. Equity may be expressed as horizontal (providing similar care for individuals with similar needs) or vertical (providing more care for individuals with higher needs) [17,18]. Because disadvantaged patients depend more on primary rather than specialty care to meet their health needs, [19] it is important to understand how primary care models perform for these patients.

Access to care has been extensively studied by Andersen and colleagues [20]. Andersen’s theoretical framework outlines characteristics influencing an individual’s access to primary care: demographics, social structure, health beliefs, enabling resources, family/community, need-perceived versus evaluated care, and health service utilization [20]. Other frameworks have since added criteria focused on other disadvantaged populations such as migrants, but the core elements have remained. The acronym PROGRESS, which stands for place of residence, race/ethnicity/culture/language, occupation, gender/sex, religion, socio-economic status, and social capital, [21] draws attention to the spectrum of social factors that stratify health opportunities and outcomes [22]. Using these frameworks, this study sought to understand the relationship between socio-economic status and health care utilization in four different delivery models.

When Canada’s universal health care system was established in the 1960s, all family physicians were paid on a Fee-For-Service (FFS) basis in which remuneration is directly tied to services rendered and payment is determined strictly by the type of service provided. Since that time, Ontario (Canada’s largest province) has introduced a number of different care models in an ongoing effort to balance cost, efficiency, efficacy, and equity in primary care delivery. In the early 1970s, Ontario set up Community Health Centres (CHC), a multidisciplinary care delivery model designed to address the needs of socially disadvantaged populations and replace more fragmented health services with comprehensive primary health care. These centres are established in disadvantaged communities and give priority care to vulnerable groups. Health professionals such as nurses and social workers work alongside family physicians, all of whom are strictly salaried, to offer a broad spectrum of services centered on their priority population (Salaried model) [23-25]. During the same era, the provincial government introduced the Capitation model (Health Service Organizations), wherein providers are compensated based on the number of patients (age and sex adjusted) they enroll [26]. The move towards Capitation models was based, in part, on the premise that separating compensation from the number of visits and services rendered would allow the physician to provide the requisite care without concern for production, presumably supporting more equitable care. A second Capitation model called the Family Health Network was introduced in 2001. Providers working in a Family Health Network receive Capitation payments as well as an additional 10% of the normal FFS amount for billed visits, and performance bonuses for achieving preventive care population targets for enrolled patients. Table 1 provides a tabular summary of the models’ characteristics.

**Table 1:** Health care payment model on Ontario

**Characteristic**	**Community health centre (CHC)**	**Fee for service (FFS)**	**Family health network (FHN)**	**Health service organization (HSO)**
Year introduced	1970s	1965	2001	1973
Group size	Group practice, size unspecified	1 Physician	Minimum 3	Minimum 3
Physician remuneration	Salary	Based on fees for services provided	Blended capitation^b^	Capitation^b^
Patient enrollment	Required, no roster size limit	Not required	Required, disincentive to enroll >2,400	Required, disincentive to enroll >2,400
Access	Extended office hours	No specified requirements	Extended office hours, THAS^c^	Extended office hours, THAS^c^
Multidisciplinarity^a^	Extensive	None	Some	Some

Table adapted from: Russell GM, Dahrouge S, Hogg W, Geneau R, Muldoon L, Tuna M: **Managing chronic disease in Ontario primary care: the impact of organizational factors.***Ann Fam Med* 2009, **7:**309–318.

^a^Multidisciplinarity refers to the presence within a practice of allied health professionals who are neither doctors nor nurses (e.g., physiotherapists, pharmacists, social workers, nurse practitioners, dieticians).

^b^Blended Capitation is a method of funding health care in which the funder provides physicians with a base payment for each patient (adjusted for age and sex) enrolled in their practice. Physicians provide comprehensive care for all patients in their panel, and receive incentives, premiums, and special payments for the provision of supplemental primary health care services.

^c^Telephone Health Advisory Service, a patient telephone advisory system for which physicians are required to provide on-call services 24 hours a day, 7 days a week.

At the time of the study, Capitation-based models, FFS clinics, and Community Health Centres serve roughly 50%, 40%, and 5% of the population of Ontario, respectively. The co-existence of these primary care models within the same geo-political environment provides an ideal opportunity to compare their impact on various measures of equity in service delivery.

The overarching objective of the current study was to assess equity in health care delivery among Ontario’s primary care practices. Specific objectives were to: 1) identify differing health care needs across socio-economic groups, 2) assess primary care service delivery across socio-economic groups within each primary care model, and 3) determine whether the size of disparities in these services are meaningfully different across organizational models.

## Methods

### Design

This evaluation is a secondary analysis of data collected in the cross-sectional study entitled Comparison of Models in Primary Care (COMP-PC), conducted in 2005–6 [27]. The study was approved by the Ottawa Hospital Research Ethics Board.

### Sample

In the original study, all known and eligible Family Health Networks (New Capitation, n = 94), Community Health Centres (Salaried, n = 51), and Health Service Organization (Established Capitation, n = 65) practices, and a randomly selected sample of the 155 eligible FFS practices were invited to participate. Eligible practices were required to have belonged to their model for a minimum of one year. Recruitment efforts continued until 35 practices in each model agreed to participate or when time constraints required recruiting to cease. Thirty two Established Capitation practices and 35 practices in all other models were enrolled. Salaried practices had belonged to their model for an average of 17 years, FFS practices for 15 years, Established Capitation practices for 16 years, and New Capitation practices for 2.3 years. Details of the study methodology and key features of the models are reported elsewhere [27]. Table 2 shows the profile of practices in the four models. The study group was determined to adequately represent the population in each model [27]. Due to the fixed sample size and multiple outcomes, power calculations were not performed.

**Table 2:** Characteristics of practices across primary care funding models

	**Primary care model**^**†**^			
	**Salaried**	**Fee for service**	**New capitation**	**Established capitation**
Characteristic*	***n* = *856***	***n* = *849***	***n* = *827***	***n* = *752***
**Patient profile**				
Age, yr, mean^^^	44	48	48	50
Female, %^^^	68	59	59	54
Insured in Ontario, %	95.3	99.8	99.9	100
> 1 visit in previous year, %^^^	87	85	80	76
*Chronic diseases*				
No. of chronic diseases per patient, mean^^^	0.33	0.35	0.40	0.44
Hypertension, %^§^	19	21	25	25
Diabetes mellitus, %	7.8	6.6	7.6	8.6
Coronary artery disease, %^§^	4.8	5.4	6.9	8.8
Congestive heart failure, %	0.8	1.4	1.4	2.0
≥1 chronic disease, %^§^	23	25	29	30
**Contextual factor**	*n* = *35*	*n* = *35*	*n* = *35*	*n* = *32*
< 10 km to hospital, %	71	85	94	87
Rurality index^¶^ ≥ 4, %^§^	69	51	86	88
**Family physician profile**	*n* = *108*	*n* = *58*	*n* = *80*	*n* = *42*
No. of years since graduation, mean^^^	19	22	23	29
Presence of ≥ 1 female family physician, **%^^^	85	49	49	25
Foreign trained, %^††^	9.3	17.2	2.5	14.3
College of Family Physicians of Canada certification, %	79	85	78	68
**Organizational structure**	*n* = *35*	*n* = *35*	*n* = *35*	*n* = *32*
No. of nurses per FTE family physician, mean^^^	0.9	0.2	0.6	0.7
Panel size < 1600 patients per FTE family physician, %^^^	85	48	58	43
Booking interval for routine visit, min, mean^^^	25	13	14	14
*Staffing*				
Solo practice, %^§^	0	26	37	38
Presence of nurse-practitioner(s), %^^^	100	8.6	31	18.8
No. of nurses, mean^^^	2.7	0.6	2.0	1.1
*Information technology*^^^^				
Electronic health records, %^§^	29	14	57	44
Electronic system for patient scheduling, %^§^	97	63	71	69
Electronic reminder system for recommended patient care (e.g., screening), %^††^	26	14	46	28
Electronic interface to external laboratory/diagnostic imaging, %^§^	46	14	51	41

Reprinted from: Dahrouge S, Hogg WE, Russell G, Tuna M, Geneau R, Muldoon LK, Kristjansson E, Fletcher J: **Impact of remuneration and organizational factors on completing preventive manoeuvres in primary care practices.***CMAJ* 2012, **184**(2):E135-E143. © Canadian Medical Association 2012. This work is protected by copyright and the making of this copy was with the permission of the Canadian Medical Association Journal (http://www.cmaj.ca) and Access Copyright. Any alteration of its content or further copying in any form whatsoever is strictly prohibited unless otherwise permitted by law.

Note: *CI* = confidence interval, *FTE* = full-time equivalent.

*Characteristics shown were obtained from chart data, provider survey data and organizational survey data and used in the analyses.

^†^The four models are known by their financing arrangement: salaried (community health centre), fee for service (fee-for-service practices), new capitation model (family health networks) and established capitation model (health services organizations). See Table 1 for more information.

^^^Characteristic is significantly different (*p* < 0.001) across the models; c2 or F test (analysis of variance [ANOVA]), as appropriate.

^§^Characteristic is significantly different (*p* < 0.01) across the models; c2 or F test (ANOVA), as appropriate.

^¶^Rurality index is based on the Rurality Index of Ontario and ranges from 0–100.

**The presence of a female family physician could only be determined from the respondents. Since at least 50% of the providers were required to participate, it is likely that some practices in which not all providers participated were wrongly coded as not having a female family physician.

^††^Characteristic is significantly (*p* < 0.05) different across the models; c2 test or F test (ANOVA), as appropriate.

^^^^For information technology factors, practices were asked to report whether the practice site had implemented, to any extent, each of the technologies listed.

### Data collection

In each of the 137 practices, 30–50 patients were surveyed sequentially in the waiting room of their clinic. Patients were eligible if they were under the care of one of the family physicians or nurse practitioners participating in the study, age 18 years or older, not severely ill or cognitively impaired, and able to communicate in English or French either directly or through a translator.

### Instruments and outcome measures

Patients completed the first part of the survey prior to their visit with their provider. That section captured detailed patient socio-economic information and elicited the patient’s response on seven dimensions of service delivery. Patients were asked to identify the highest level of education they had attained. Individuals who had not completed a high school education were coded as “low education”; all others were coded as “average education”.

Income status was determined using Low Income Cut Offs (LICO) as a benchmark measure. LICO are calculated by Statistics Canada and represent a threshold beyond which necessary expenses become a significant financial burden. Families who earn an annual income below LICO must spend over 20% more of their total wages on basic necessities (i.e. food, shelter, and clothing) than do Canadian families earning the national average income [28]. As costs vary based on geographic location and family size, LICO are adjusted based on the number of family members in the household and the population of the municipality in which they reside (See Table 3). Since our data included income ranges and not exact figures, we used the upper limit of each range as an income value. We used practice addresses in place of patient addresses to determine the population of each patient’s municipality. Patients whose incomes fell below their LICO were classified as low income, while those whose incomes were above their LICO were classified as high income. High income does not constitute a homogenous group, but rather represents a broad cross-section of individuals who, though of various means, are not burdened by the cost of essentials such as food and shelter to the same extent as low income individuals.

**Table 3:** Statistics Canada low income cut-offs (before tax)

	**Population of area of residence**				
**Family size (#individuals)**	**Rural (farm and non-farm)**	**Small urban regions**	**30,000 to 99,999 individuals**	**100,000 to 499,999 individuals**	**500,000 or more individuals**
1	$14,303	$16,273	$17,784	$17,895	$20,778
2	$17,807	$20,257	$22,139	$22,276	$25,867
3	$21,891	$24,904	$27,217	$27,386	$31,801
4	$26,579	$30,238	$33,046	$33,251	$38,610
5	$30,145	$34,295	$37,480	$37,711	$43,791
6	$33,999	$38,679	$42,271	$42,533	$49,389
7+	$37,853	$43,063	$47,063	$47,354	$54,987

**Adapted from:**http://www12.statcan.ca/census-recensement/2006/ref/dict/tables/table-tableau-18-eng.cfm.

Categorization of Low Income Cut Off (LICO) based on size of community in which the household resides, the number of individuals in the household, and the total income for the household.

Health service delivery questions were adapted from the Primary Care Assessment Tool-Adult Edition (five scales), [29,30] supplemented with a Humanism scale and a Trust scale [31,32]. Scales were also made available in French [33]. Each of the Primary Care Assessment Tool scales contained three or four questions pertaining to care quality (e.g. “when you need a regular general check-up, do you go to your doctor before going somewhere else?”) scored on a four-point Likert scale. The Primary Care Assessment Tool has been validated as congruent with its theoretically-derived measures. We report the scores for the overall Primary Care Assessment Tool health service delivery questions and the individual scale scores separately. The supplementary Humanism scale consisted of eight questions on a seven-point Likert scale and the supplementary Trust scale contained ten questions on a five-point Likert scale.

Patients completed the second part of the survey immediately after visiting their care provider. This section captured information relating to the encounter they just had, including self-reported visit duration and a measure of health promotion. Health promotion was assessed by asking whether any of six healthy lifestyle subjects were discussed at that encounter. These six items were based on the recommendations of the Canadian Task Force on Preventive Health Care [34,35] and responses were elicited using the following question: “In today’s visit to your clinic were any of the following subjects discussed with you? (yes/no/don’t know)”.

### Analysis

#### Objective 1 – health care needs across socio-economic groups

We compared the profile of patients across socio-economic groups using Chi square and Analysis of Variance, as appropriate, to identify differences in health status and infer health care needs for different patient groups. The four indicators of health status are identified in Table 4.

**Table 4:** **Percentage distribution of selected characteristics by delivery model**, **sex**, **age**, **English language ability**, **rurality**, **employment**, **and self**-**reported health status**

**Info type**^**a**^	**Characteristics**	**High income & education**	**Low income**^**b**^	**Low education**^**c**^	**Low income & education**
	**Sample size (number of patients)**^**d**^	3010	444	386	215
	Community Health Centres – Salaried	509 (58%)	194 (22%)	75 (9%)	93 (11%)
	Fee-For-Service – Fee-For-Service	824 (79%)	88 (8%)	92 (9%)	43 (4%)
	Family Health Teams – New Capitation	920 (79%)	85 (7%)	108 (9%)	46 (4%)
	Health Service Organizations – Established Capitation	757 (77%)	77 (8%)	111 (11%)	33 (3%)
	**Socio-demographic and social disadvantage profile**				
Demog	Sex (women)*	64%	75%	54%	72%
Demog	Age (mean years)*	48	44	60	63
Demog	Rurality index (mean)*	13	12	16	16
Demog	Distance from home to practice > 10 km*	26%	20%	22%	20%
SD	Not speaking English or French at home*	0.8%	4.5%	1.6%	3.3%
SD	Aboriginal*	1.1%	3.2%	1.0%	1.9%
SD	Uninsured (in Canada)*	0.9%	4.5%	1.6%	3.7%
SD	Unemployed*	2.1%	16.9%	3.6%	15.8%
SD	Recent immigrant (< 5 years)*	1.1%	6.2%	0.5%	3.8%
	**Health status**				
H	Mean days with poor mental*/physical* health in past 30 days	3.9/4.6	7.8/7.9	4.0/6.8	7.0/9.4
H	Mean days limited by poor mental or physical health in past 30 days*	3.3	6.9	5.4	7.5
H	Self-perceived health very good-excellent*	88%	64%	73%	60%
H	Presence of at least one chronic disease*/Mean number of chronic diseases*^e^	69%/1.6	77%/2.2	84%/2.6	88%/2.9
	**Relationship with the practice**				
	Provider is a Nurse Practitioner *	2.5%	4.1%	1.7%	3.5%
	Seeing their own provider at that visit	94%	92%	96%	93%
	Attending the practice for more than 2 years*	85%	79%	84%	83%
	Number of visits to the office in previous year (mean*, median)	5.4/4	10.0/6	7.7/5	9.0/6
	Main reason for visit – Chronic (long term) problem*	26%	42%	38%	39%

^a^Info Type column identifies the category of information adjusted for in the analyses for the given row.

Socio-demographic information = Demog.

Health status = *H*.

Social disadvantage = *SD*.

^b^Low income = individuals living under the Low Income Cut Off, as defined by Statistics Canada.

^c^Low education = less than high school degree.

^d^4,164 and 5,113 individuals provided income and education data, respectively.

^e^13 chronic diseases assessed (self-reported).

Statistically significant differences (p < 0.05) are identified by “*”.

#### Objective 2 – equity of primary care delivery within models

For each model, we compared the quality of care for individuals with low income/education to those with high income/education using multi-level linear or logistic regressions, as appropriate. The SPSS 18 mixed model procedure and the Glimmix procedure in SAS were used to account for the clustered nature of the data. Practice variables, including summary provider characteristics made up level two, and patient variables made up level one.

Our primary analyses sought to determine whether individuals in the low income and low education groups received different levels of care compared to those in the high income and education groups. In these analyses, the quality of care measures (shown in Tables 5) were the dependent variables and socio-economic status was the main independent variable. In the main analyses, adjustments were made for demographic factors: patient age, sex, rurality index score, and travel distance to the practice (identified as “demog” in Table 4). The analyses were performed in each of the four primary care models separately. Linearity of continuous variables was verified, and these variables were transformed or categorized as necessary. The estimated beta values from the regressions assessing the “number of yearly visits” were used to estimate the number of visits for the typical patient: a woman, between the ages of 30–65, living in a non-rural region and travelling less than 10 km to the nearest hospital.

**Table 5:** **Health service delivery across socio**-**economic groups and between practice models**

	**Salaried**	**Fee-for-service**	**New capitation**	**Established capitation**
**Duration of visit**				
Overall mean (minutes)	*24*	15	15	15
Estimated effect – Beta (95% CI)^a^				
*Low income*^b^	3.1 (-0.7, 7.0)	1.1 (-1.3, 3.4)	0.5 (-1.8, 2.8)	-0.3 (-2.6, 2.0)
*Low education*^c^	-1.3 (-7.1, 4.5)	-0.8 (-3.1, 1.5)	0.1 (-2.0, 2.3)	-0.1 (-2.1, 1.9)
*Low income and education*	0.2 (-4.9, 5.3)	0.7 (-2.7, 4.2)	1.0 (-2.1, 4.1)	0.1 (-3.4, 3.6)
**Number of visits per year**				
Overall mean (# visits)	8.3	7.2	5.3	4.8
Estimated effect – Beta (95% CI)^a^				
*Low income*	***7.0* (*4.8***, ***9.2*)**	**3.5 (1.6**, **5.3)**	**1.4 (0.2**, **2.6)**	**1.6 (0.5**, **2.6)**
*Low education*	**3.6 (0.3**, **6.9)**	***4.2 *** **(*2.3***, ***6.0*)**	**1.1 (0.0**, **2.3)**	**1.2 (0.3**, **2.2)**
*Low income and education*	***5.4 *** **(*2.4***, ***8.4*)**	**3.7 (1.0**, **6.3)**	0.9 (-0.7, 2.5)	**1.7 (0.1**, **3.3)**
**Estimated yearly visits for the typical patient**^**d**^				
*No risk factor*	7.0	7.3	5.8	4.9
*Low income*	14.0	10.7	7.2	6.5
*Low education*	10.6	11.4	7.0	6.2
*Low income and education*	12.4	10.9	6.7	6.6
**Primary care assessment tool – Overall**^**e**^				
Mean overall score	86%	86%	86%	88%
Estimated effect – Beta (95% CI)^a^				
*Low income*	-0.6 (-2.2, 1.1)	0.0 (-2.0, 2.0)	-0.8 (-2.8, 1.2)	1.0 (-1.0, 2.9)
*Low education*	0.5 (-1.9, 2.9)	1.8 (-0.2, 3.8)	-0.5 (-2.3, 1.4)	0.6 (-1.1, 2.3)
*Low income and education*	1.5(-0.6, 3.7)	**3.9 (1.0**, **6.7)**	**3.0 (0.4**, **5.7)**	2.1 (-0.8, 4.9)
**Primary care assessment tool - Individual scales**^**f**^				
Effect of low income and low education– Beta (95% CI)^a^				
First Contact Accessibility	-2.3 (-6.1, 1.6)	**8.7 (3.7**, **13.8)**	1.7 (-3.0, 6.5)	3.1 (-1.3, 7.4)
First Contact Utilization	-1.2 (-3.1, 0.6)	-1.1 (-3.2, 1.0)	0.3 (-2.0, 2.6)	0.6 (-1.7, 2.9)
Cultural competency	2.2 (-1.3, 5.8)	2.4 (-2.6, 7.4)	3.4 (-1.5, 8.2)	1.3 (-4.6, 7.1)
Family Centered Care	2.5 (-0.3, 5.2)	2.9 (-1.1, 6.9)	**4.4 (0.8**, **7.9)**	2.3 (-2.0, 6.5)
Relational Continuity	**5.1 (1.8**, **8.5)**	**5.1 (1.3**, **9.0)**	**5.3 (1.5**, **9.2)**	2.0 (-1.9, 5.9)
Humanism	-0.5 (-3.3, 2.3)	1.8 (-1.9, 5.5)	**4.9 (1.1**, **8.7)**	1.8 (-2.6, 6.2)
Trust	-0.1 (-2.8, 2.7)	1.7 (-2.0, 5.5)	2.3 (-1.3, 5.9)	-0.2 (-4.1, 3.8)
**Health promotion - Discussed at least one subject**^**g**^				
*Low income*	1.3 (0.6, 1.8)	1.1 (0.7, 1.8)	1.3 (0. 8, 2.1)	1.2 (0.6, 1.8)
*Low education*	0.7 (0.4, 1.2)	0.9 (0.6, 1.5)	1.1 (0.7, 1.7)	0.9 (0.6, 0.4)
*Low income and education*	**1.7 (1.0**, **2.8)**	0.8 (0.4, 1.7)	1.2 (0.6, 2.2)	0.9 (0.4, 2.0)
**Discussed healthy foods**^**g**^				
*Low income*	1.3 (0.9, 1.9)	0.8 (0.4, 1.5)	1.0 (0.5, 1.8)	0.8 (0.4, 1.6)
*Low education*	0.7 (0.4, 1.4)	0.7 (0.4, 1.4)	0.8 (0.5, 1.5)	1.1 (0.6, 1.9)
*Low income and education*	1.4 (0.8, 2.3)	1.0 (0.4, 2.5)	1.1 (0.5, 2.3)	0.5 (0.1, 1.7)
**Discussed home safety**^**g**^				
*Low income*	1.3 (0.6, 2.7)	3.9 (1.4, 10.5)	**6.7 (2.1**, **21.2)**	1.0 (0.2, 4.4)
*Low education*	1.0 (0.3, 3.5)	**4.9 (1.9**, **12.7)**	2.8 (0.8, 10.2)	0.9 (0.3, 3.2)
*Low income and education*	**2.4 (1.1**, **5.5)**	2.2 (0.5, 10.7)	1.9 (0.2, 16.3)	2.0 (0.4, 9.8)
**Discussed family conflict**^**g**^				
*Low income*	1.2 (0.8, 2)	**2.7 (1.3**, **5.4)**	1.7 (0.9, 3.3)	**2.1 (1.1**, **4.2)**
*Low education*	1.0 (0.5, 2.3)	1.6 (0.7, 3.5)	1.0 (0.4, 2.4)	0.6 (0.3, 1.5)
*Low income and education*	1.4 (0.7, 2.7)	1.7 (0.5, 5.2)	**2.3 (1.0**, **5.4)**	0.7 (0.2, 3)
**Discussed exercise**^**g**^				
*Low income*	1.2 (0.8, 1.7)	0.9 (0.6, 1.6)	1.0 (0.6, 1.8)	0.7 (0.4, 1.3)
*Low education*	0.5 (0.3, 1.0)	0.7 (0.4, 1.2)	0.9 (0.6, 1.5)	0.7 (0.4, 1.3)
*Low income and education*	0.7 (0.4, 1.2)	1.2 (0.6, 2.3)	0.9 (0.4, 2.1)	1.0 (0.5, 2.1)
**Discussed smoking**^**g**^				
*Low income*	0.8 (0.5, 1.3)	1.8 (1.0, 3.2)	1.0 (0.5, 2.0)	1.3 (0.7, 2.4)
*Low education*	1.2 (0.5, 2.5)	1.3 (0.7, 2.5)	1.6 (0.9, 2.9)	0.7 (0.3, 1.4)
*Low income and education*	1.7 (0.9, 3.0)	1.4 (0.6, 3.5)	2.1 (1.0, 4.6)	1.2 (0.5, 3.4)
**Discussed alcohol**^**g**^				
*Low income*	0.8 (0.5, 1.4)	0.3 (0.1, 1.3)	0.4 (0.1, 1.1)	1.3 (0.6, 2.9)
*Low education*	0.4 (0.1, 1.1)	0.6 (0.2, 1.6)	0.9 (0.5, 1.9)	0.6 (0.2, 1.5)
*Low income and education*	0.7 (0.3, 1.5)	2.1 (0.7, 6.4)	0.8 (0.3, 2.4)	2.5 (0.9, 7.4)
**Overall mean frequency of discussing at least one subject**^**h**^	1.19	0.83	0.93	0.84

**Bolded numbers** = Statistically significant (p < 0.05) difference across socio-economic groups.

*Italics* = Statistically significant (p < 0.05) difference between practice models.

^a^Individuals living above the *LICO* and with at least a high school education make up the reference category. The model was adjusted for socio-demographic factors only (Demog in Table 4).

^b^Low income is defined as falling below *LICO* (Table 3). The average income group makes up the reference category, and includes all patients with annual incomes above the *LICO*.

^c^Low education is defined as not having completed high school. The average education group makes up the reference category, and includes all patients with secondary school diplomas.

^d^Derived from regression betas. A typical patient is a woman, ages 30–65, living in a non-rural region, where travel distance to the nearest hospital is less than 10 kilometres.

^e^Summary score for first contact accessibility and utilization, cultural competency, family centered care, and ongoing care/relational continuity.

^f^The effect sizes for individuals living below *LICO* and with low education only are shown. The effect sizes for individuals with low income only and or low education only did not exceed 3% in either direction (results now shown).

^g^The Odds Ratio of having discussed that subject across a socio-economic group.

^h^Overall mean frequency of discussing any one of the subjects included in the analysis during any visit within the study’s parameters.

Definitions: *First contact accessibility* is the ability to obtain patient-initiated needed care from the provider of choice within a time frame appropriate to the urgency of the problem; *first contact utilization* is the extent to which the provider/practice is first used for various types of problems; *cultural competency* is the extent to which providers integrate cultural considerations into communication, assessment, diagnosis and treatment planning; *family*-*centered care* is the extent to which providers consider the family (in all its expressions), understand its influence on a person’s health and engage it as a partner in ongoing health care; *relational continuity* is a therapeutic relationship between a patient or client and one or more identified providers that spans separate health care episodes and delivers care that is consistent with the patient’s or client’s biopsychosocial needs; *humanism* is an approach to medicine that emphasizes the relationship between caregiver and patient; *trust* is the degree to which patients or clients believe that their provider will care for patients’ or clients’ best interests (adapted from Haggerty et al.) [36].

To determine whether observed differences across socio-economic groups were explained by differences in factors reflecting social disadvantage (identified as SD in Table 4) or by differing health status between the groups (identified as H in Table 4), we conducted a second and third set of analyses in which, alternately, these factors were added to the equation.

#### Objective 3 – assess equity of primary care delivery between models

We used t-statistics to compare the effect size (absolute estimated beta values) of the socio-economic variables derived from the regression models in Objective 2 to determine whether the size of the observed disparities across socio-economic groups differed between primary care models.

## Results

### Characteristics of the study population

Seventy nine percent (5,361) of patients approached completed the survey. Amongst these, 4,166 and 5,113 provided sufficient information to determine income and education levels respectively, and 4,055 (76%) provided both. Individuals who did not report their income had a profile that was more consistent with higher income individuals (data not shown). The numbers of patients in each practice model and socio-economic group are listed in Table 4.

### Objective 1

Compared to individuals with high income and education, those with low income were more likely to be women, unemployed, recent immigrants, and in poorer health. Those without a high school education were more likely to be men, older, and to report lower health status on some indicators. On most health indicators, people with low income and education had the lowest health status (Table 4).

### Objectives 2 & 3

#### Disparities in performance measures

The average encounter duration was 24 minutes in the Salaried model and 15 minutes in the other models, with no statistically significant differences in visit length across socio-economic groups within a model (Table 5). After adjusting for demographic factors (age, sex, rurality and distance from hospital), compared to individuals with high income and education, those with low income and/or education reported substantively more visits in the previous year in all models, although to a lesser extent in the Capitation models (Table 5). The number of additional visits ranged from 3.6-7.0 in Salaried, 3.5-4.2 on FFS, and 0.9-1.7 in Capitation. The number of additional visits was significantly higher for individuals of low income receiving care in the Salaried model (7.0, 95% confidence interval (CI): 4.8, 9.2) compared to other models (< 4), for individuals with low education in the FFS model (4.2, CI: 2.3, 6.0) compared to the Capitation models (< 2), and for individuals with low income and education receiving care in the Salaried model (5.4, CI: 2.4, 8.4) compared to the Capitation models (<2).

The estimated number of yearly visits for the typical patient with low income and/or education was 11–14 for the Salaried model (vs. 7, no risk factor), 11 for the FFS model (vs. 7, no risk factor), and 6–7 for the two Capitation models (vs. 5–6 for no risk factor) (see Table 5). Adjusting for other social disadvantage features (SD in Table 4) had little impact on effect size (estimated beta values), and adjusting for health status (H in Table 4) diminished effect size only slightly (*results not shown*).

#### Health service delivery scales

The overall Primary Care Assessment Tool score showed small differences (<5%) between risk groups and models (Table 5). Adjusting for health factors or other social disadvantage factors did not have an apparent impact on effect sizes (results not shown). An analysis of the seven individual scales showed that individuals with both risk factors (i.e. low income and low education) did not report lower performance levels, and in some instances, reported higher scores than those with neither risk factor (Table 5). Relational continuity was higher for these at-risk individuals in the Salaried, Fee-For-Service, and New Capitation models. Family centered care and Humanism were also higher for this group but only in the New Capitation model. The largest difference was observed in first contact accessibility in the Fee-For-Service practices (beta = 8.7%, 95% CI, 3.7%, 13.8%). The size of the disparity in these measures was not statistically significant across models. Adjusting for health and social disadvantage yielded similar results.

#### Health promotion

The odds of having discussed at least one healthy lifestyle subject with a physician were significantly higher for individuals with both risk factors in the Salaried model only (Odds Ratio = 1.7, CI: 1.0, 2.8) (Table 5). This effect was lost when other social disadvantage factors were included in the question (Odds Ratio (CI): 1.0 (0.7, 1.5) (*results not shown*)). The odds ratio of having discussed each individual lifestyle subject is also shown in Table 5. Home safety and/or family conflict were more likely to be discussed in some socio-economic groups in most models (see Table 5). There were no significant differences in disparities across models.

## Discussion

This study found that some measures of primary care services were significantly higher for people who are disadvantaged by low income and/or education. This finding was true across all payment models. However, whether this was sufficient to achieve vertical equity (i.e. to meet the greater demands of these more vulnerable patients) isn’t clear.

Socio-economically disadvantaged patients were more likely to be women, have poorer health, be new immigrants, and speak a language other than English or French at home. These factors may cause providers to spend more time on, or have more frequent encounters with, certain patients, making it more difficult for them to provide quality care [37]. We observed what appeared to be accommodation for greater perceived needs in the way of additional visits in all models. These findings correspond with previous studies demonstrating greater service use in patients with socio-economic risk factors relative to individuals without those risk factors [4,38-40]. However, the pattern of realized access and the extent to which accommodations were made for individuals with higher needs varied significantly between models. Patients receiving care in the Salaried model had significantly longer visit durations (24 minutes) than those receiving care in FFS or Capitation practices (15 minutes). We found a correlation between risk and number of yearly visits in all models, although to a lesser extent in the Capitation models where the number of yearly visits to the practice is already lower. As a result, the total yearly encounter duration for an individual with low income and low education is on average 298 minutes in the Salaried model, 164 minutes in FFS, and approximately 100 minutes in Capitation models. Our findings are in keeping with the anticipated influence of payment model on work patterns [41].

In Salaried model, which is designed to serve more vulnerable populations, the salaried structure removes the financial barrier that providers encounter when accommodating patients who have greater needs. This is reflected in our measures of encounter duration (a measure of realized access), but not in our measures of perceived access such as the accessibility score or overall score on PCAT self-reported measures. This is potentially due to higher patient expectations in that model. Since spending more time on individual patients naturally reduces the total number of patients that a physician can manage, concerns about the Salaried model’s efficiency have been raised. However, a recent comparison of primary care models in Ontario concluded that, after accounting for patient profile, patients receiving care at the Salaried model had a considerably lower rate of emergency room visits than would be expected. Furthermore, a 2013 study found that Salaried models of care delivery were positively associated with better technical quality of care [42]. One potential interpretation of this would be that more intense patient management may be effective in mitigating poor outcomes [43]. A comprehensive economic evaluation is required to fully capture the societal benefit of the Salaried model.

We found evidence of accommodation for patients with greater needs in the number, but not duration, of visits across all models. This was most prominent in the Salaried and Fee-For-Service models. There is evidence that family physicians’ practice patterns depend on the way they are paid [44,45]. Jegers et al. suggest that a “variable” payment system that is linked to services rendered (i.e. FFS) incentivizes the delivery of services, potentially resulting in overproduction and unnecessary care, whereas a fixed remuneration system in which the payment is dissociated from the number of services (i.e. Capitation) allows the provider flexibility both in the encounter duration and the number of encounters, but can lead to compromised necessary care due to a financial incentive to limit the duration and number of visits provided to existing patients in favour of enrolling new patients [46]. While we cannot comment on whether all services rendered in the FFS model are required, we can conclude that accommodation for higher needs is likely achieved. However, while the profile of patients was similar in both models, the total encounter time for individuals with low income and education was more than 50% longer in FFS than in Capitation. We also found smaller accommodation in the number of visits for patients with higher needs in the Capitation model. Capitation is the predominant payment model in Ontario, as well as the model most likely to serve vulnerable populations, making the lower rate of visits among disadvantaged groups a growing concern [47]. However, this study cannot determine whether Capitation’s lower encounter times and less frequent encounters among vulnerable groups represent improved efficiency or reduced accessibility. Compared to other models, there was no evidence that these shorter visit times led to lower quality provider-patient relationships. However, if one believes that every encounter is an opportunity to provide healthy lifestyle counselling, then there are comparatively fewer such opportunities among capitated practices. In Ontario, adjustments for Capitation payment are based solely on the age and sex of patients and do not take into account the often greater care needs of patients with lower socio-economic statuses. Therefore, the payment system does not support the adequate care of more complex patients [48]. Creation of more complex formulas for capitation payments and blended-fee structures, although difficult, may reduce physician behaviours that adversely affect equitable service delivery [49].

Overall ratings of all health service delivery measures were high in all groups and models, with no evidence of compromise for those who are socio-economically disadvantaged. In fact, relational continuity was better for disadvantaged people across most models. However, despite evidence that socio-economically disadvantaged people have higher health risk behaviours such as smoking, excessive alcohol consumption, and poor eating habits, [7,8] and the fact that improved accessibility to health promotion could reduce these risks, [50] we found no consistent evidence that individuals with low income/education were more likely to receive healthy lifestyle counselling addressing these factors in any of the models studied.

Our study did not account for the total number of times health promotion items were discussed over a prolonged period, but instead measured the likelihood of the discussion taking place at the patient’s last visit. While the former would have allowed us to measure the rate or density of health promotion, the latter allowed us to evaluate whether practices adhered to the Canadian Task Force on Preventive Health Care’s recommendation that health promotion be discussed at every patient encounter, which we ultimately considered more valuable.

### Limitations

This study has several limitations. Because the sample size was fixed, some negative findings could be related to inadequate power and should be interpreted cautiously. While the patient participation rate was good (79%), a significant portion of potential respondents (22%) did not provide the information required to determine their income status. If these individuals differed from the respondents in their experience of primary care, this study would not have accurately represented the disparities across socio-economic groups. We were required to estimate patients’ area of residence by using the address of the practice they attended in place of their actual home address. This may not be an accurate way of estimating residential addresses, particularly in Ontario where physician shortages mean that patients don’t always have the choice of enrolling with a provider near their home [51]. The study focused solely on care provided during patient visits, ignoring phone visits, referrals, and visits made outside of the clinic (e.g. home care, outreach). The varying incentives provided by Capitation versus FFS billing could conceivably lead to different approaches to these types of external care. The study was conducted only four years after Family Health Networks (the New Capitation model) were introduced. While all practices were required to have operated in that model for at least one year, it is possible that providers took longer to adapt their behaviour to new circumstances. In this case, the results pertaining to the New Capitation model may not reflect the practices in their present, more established state. The distribution of the socio-economic factors considered in this study adequately reflects that of the general Ontario population. As a result, risk categories had few patients, limiting our ability to measure differences in their care experience. Health service delivery scale scores were high across all groups and models, potentially suffering from a ceiling effect, and further limiting our ability to detect meaningful differences across groups. Most of the outcomes we used are process measures and thus our results should not be extrapolated to clinical outcomes. Our study does not address relative changes in health status over time or unforeseen events such as emergency room visits, avoidable admissions and preventable deaths. This study should be considered as exploratory as it conducted multiple comparisons and the results are at high risk of type one error. This study focuses on the association between different clinical payment models and equity of care delivered. It did not seek to identify the impact of factors that impact provider capacity such as case mix, panel size, and availability of neighbourhood services, on the provider’s ability to provide equitable care. The Primary Care Assessment Tool used in this study relies on self-reported data, and as such is potentially vulnerable to recall bias. Lastly, the measures of access may also have been influenced by the desires of patients with conflicting schedules (e.g. prohibitive work schedules), limiting our ability to establish whether differences across socio-economic groups are driven by the model rather than in differences in the patients’ care-seeking behaviour.

## Conclusions

In its most recent report, the World Health Organization described its efforts to promote the universality of care around the world [52]. The introduction of universal healthcare under the Canadian Act has reduced disparities in access to primary health care, [3] but as the system continues to evolve, policymakers need to be vigilant about the impact of new transformations on populations vulnerable to poor access and poor health outcomes. This study provides the first account of the experience of socio-economically disadvantaged individuals across different primary care organizational models. The results suggest that the remuneration structure may affect provider behaviour in a way that can influence equity. Further assessment of the impact of primary care reforms on equity in Canada is clearly required. Additionally, studies evaluating the impact of contextual factors such as neighbourhood services or deprivation level on the provider’s ability to deliver equitable care would contribute to a better understanding of the factors that influence this subject.

## Abbreviations

CI: Confidence interval; COMP-PC: Comparison of models in primary care; FFS: Fee-for-service; H: Health status; LICO: Low income cut off; SD: Social disadvantage.

## Competing interests

The authors declare that they have no competing interest.

## Authors’ contributions

RAD helped in the design of the analysis and interpretation of the results and was involved in revising the manuscript. WH conceived and led the original Comparison of Models study. WH and BK were co-supervisors of SD’s PhD thesis, which included the present work, and assisted with the interpretation of the analysis and critically reviewed the different versions of the manuscript. All authors read and approved the final manuscript.

## Pre-publication history

The pre-publication history for this paper can be accessed here:

http://www.biomedcentral.com/1472-6963/13/517/prepub
